# Levels of Lipid Parameters in Children with Arterial Ischemic Stroke and Headache: Case-Control Study and Meta-Analysis

**DOI:** 10.3390/brainsci11040417

**Published:** 2021-03-26

**Authors:** Beata Sarecka-Hujar, Joanna Sordyl, Ewa Małecka-Tendera, Ilona Kopyta

**Affiliations:** 1 Department of Basic Biomedical Science, Faculty of Pharmaceutical Sciences in Sosnowiec, Medical University of Silesia in Katowice, Kasztanowa Str 3, 41-200 Sosnowiec, Poland; 2 Department of Paediatrics and Paediatric Endocrinology, School of Medicine, Medical University of Silesia, 40-752 Katowice, Poland; joanna.sordyl@wp.pl (J.S.); etendera@sum.edu.pl (E.M.-T.); 3 Department of Pediatric Neurology, Faculty of Medical Sciences in Katowice, Medical University of Silesia in Katowice, Medykow Str 16, 40-752 Katowice, Poland; ilonakopyta@autograf.pl

**Keywords:** lipid levels, dyslipidemia, children, arterial ischemic stroke, headache

## Abstract

*Background:* Abnormalities in levels of lipid parameters are one of the main causes of cardiovascular and cerebrovascular disease in adults. There are limited data on the role of disturbances of lipid metabolism in the etiopathogenesis of arterial ischemic stroke (AIS) in children and the results provided are ambiguous. The aim of the study was to compare the levels of lipid parameters (total cholesterol [TC], triglycerides [TG], high-density lipoprotein [HDL] and low-density lipoprotein [LDL]) between children with AIS, children with headache and healthy children. In addition, we performed meta-analysis of available data on lipid parameters in young patients with AIS. *Methods*: We retrospectively analyzed 218 children hospitalized between 2002 and 2018 in the Upper-Silesian Child’s Health Center (*n* = 82 children with AIS, *n* = 45 children with headache, *n* = 91 healthy children) with available data on lipid levels, i.e., TC, TG, and HDL. The levels of LDL, non-HDL cholesterol, and a very-low density lipoprotein (VLDL) were calculated. The ratios of TC/HDL, TG/HDL and LDL/HDL were also assessed. Data between cases and controls were analyzed using STATISTICA 13.0 whereas meta-analysis was performed with RevMan version 5.4 software. *Results*: Children with headache were significantly older than children with AIS (*p* = 0.001). Ten percent of children with AIS had posterior stroke. The mean TC level was significantly higher in the AIS children than in controls or in children with headache. Mean TG and VLDL levels were significantly different between all groups (*p* < 0.001 each). The hypertriglyceridemia was more prevalent in AIS children than in children with headache (39% vs. 13%, OR = 4.16 95% CI 1.58–10.94, *p* = 0.004). Similarly, the frequency of dyslipidemia was higher in children with AIS compared to children with headache (38% vs. 22%, OR = 2.13 95% CI 0.93–4.89, *p* = 0.078). The meta-analysis was conducted based on data from 4 studies (3 studies published previously plus the results we obtained in the present case-control analysis) with total number of 236 young patients with AIS and 272 healthy controls. Significant Standard Mean Difference (SMD) was found in triglycerides level between young patients with AIS and controls (0.78 95%CI 0.30–1.26 *p* = 0.002). *Conclusions*: Lipid abnormalities, especially levels of triglycerides, seem to be of particular importance in children with AIS, as confirmed in meta-analysis. The results of the present study may be a significant contribution to the further research on the role of lipid metabolism disorders in the development of childhood stroke.

## 1. Introduction

Arterial ischemic stroke (AIS) is a relatively rare disease in children. According to literature, the incidence of ischemic stroke in childhood is about 3 cases per 100,000 children per year [1]. Pediatric AIS still remains a serious medical problem. Detrimental outcomes include seizures, recurrent strokes or even death [1,2]. The pathophysiology and the impact of potential risk factors of childhood AIS are still unclear. The most commonly reported AIS risk factors are arteriopathies, cardiac diseases, thrombophilia, metabolic diseases, traumas or infections however still approximately one third of all cases are cryptogenic [1,2]. Ischemic stroke in adults and in the group of pediatric patients is characterized by different pathophysiological mechanisms. Atherosclerosis plays the most important role in the cerebral stroke development in adults whereas thrombotic vascular occlusion in pediatric AIS is caused by endothelial and blood coagulation disorders, that lead to procoagulant states [1,2,3,4,5,6]. While AIS is most often provoked by both genetic and environmental risk factors, the role of exposure to external risk factors like lifestyle (that plays a very important role in adults), seems to have minor clinical importance in children [1,2,3,4,5,6,7,8].

In turn, headache is a very common disorder in the pediatric population. The occurrence of headache correlates with age—the older the children, the higher the frequency of headache [9]. Headache was established in 58% of children in a study based on a large group of pediatric participants (*n* = 80,876) [9]. In adults, migraine with aura was observed to be a risk factor for AIS in relatively young patients aged 15–50 years, especially in women [10].

Dyslipidemia may play a role in the pathogenesis of cerebrovascular disorders in children, especially in the pre-puberty period and in late adolescence when lipid levels are naturally highest [11,12]. Dyslipidemia was also suggested to be an important risk factor for AIS related to steno-occlusive arteriopathy [13]. Abnormalities in lipid levels were found to be a common problem in healthy children from Brazil as well as South Korea [14,15]. Such lipid disturbances in childhood may be related to a family history of dyslipidemia or premature cardiovascular disease secondary to it. Data on lipid abnormalities in headache among children are scarce [16].

We have hypothesized that as both AIS and headache have vascular origin, the evaluation of lipids levels and the lipid ratios between the two subgroups of children as well as comparison to healthy peers, is important for explanation of the etiology of these two conditions.

The aim of the present study was to compare the lipid levels as well as lipid ratios between children with AIS, children with headache and healthy children. In addition, we performed meta-analysis of available data on lipid parameters in young patients with AIS.

## 2. Materials and Methods

### 2.1. Study Participants

We retrospectively analyzed 218 children hospitalized between 2002 and 2018 in the Upper-Silesian Child’s Health Center. Children were recruited at the Department of Pediatric Neurology (within two projects: 3PO5E 135 23, N 406 037 31/0986) and the Department of Pediatric Endocrinology of the Medical University of Silesia in Katowice (Poland) (within two projects: KNW-2-050/D/5/N, KNW-2-O75/D/6/K). Preliminary data were previously published as pilot studies [17,18].

The total group of analyzed children was divided into the following 3 subgroups according to clinical presentation: children with AIS (*n* = 82), children with headache (*n* = 45) and healthy children (*n* = 91). Patients were recruited consecutively from the database.

The inclusion criteria for patients with AIS were as follows: (a) diagnosis of AIS in the acute phase confirmed by clinical and radiological criteria (computed tomography [CT] and/or magnetic resonance imaging [MRI]); (b) the age of the patients at the acute phase of stroke from 1 month to 18 years old; and (c) available data on lipid levels. Children were excluded from the stroke group in case of (a) a lack of CT and/or MRI results confirming the diagnosis, (b) head injury as a cause of AIS, and (c) lack of data on lipid levels. In some cases, the CT examinations were performed with 16-row scanner TOSHIBA AquilionS, using spiral technique, and the diagnostic protocols were adjusted the children’s age. In turn, all children with AIS underwent MRI examination using a 1.5T MR imaging system Optima 450 w GEM (General Electric, GE Healthcare). Imaging standard protocol included T1, T2, T2 Flair, susceptibility weighted image (SWI) and diffusion weighted image (DWI) sequences. MR angiography was performed using time of flight (TOF) technique which allows visualization of vessels without intravenous contrast injection. T1 3D post contrast sequences were performed in doubtful cases. The dose of gadolinium contrast was 0.1 mL/kg of body weight. DWI was the most important sequence in stroke patient as abnormalities in water diffusion in brain tissue change signal as early as 30 min after stroke incident.

The inclusion criteria for the headache group were as follows: 7–18 years old, primary headaches (migraine/tension-type headaches) for at least 6 months (min. 2 episodes/week). Patients from the headache group were excluded in case of: secondary headaches (brain tumors and other reasons for intracranial hypertension like inflammatory central nervous system diseases and/or hydrocephalus, fever, sinusitis or glaucoma), acute respiratory tract or gastrointestinal infection, hormonal disorders (hypothyroidism or diabetes), acute/chronic disorders with hypertension/lipid disturbances (renal and heart diseases, children born small for gestational age or familial hyperlipidemia).

The control group consisted of healthy children with no vascular disorders who were hospitalized due to benign, uncomplicated head injury or familial short stature.

The study was conducted in accordance with the Declaration of Helsinki, and the Ethics Committee of the Medical University of Silesia in Katowice approved the study (KNW/0022/KB/88/18 and KNW/0022/KB1/67/14, KNW/0022/KB1/67/I/14/16). To the present research no new patients were recruited since the analyses are based on the previously obtained data, therefore no written consents were needed.

### 2.2. Lipid Levels

In this study, we retrospectively analyzed levels of lipids which were previously measured spectrophotometrically with enzymatic methods using commercial kits. The following lipid parameters were taken into account: total cholesterol (TC), triglycerides (TG), and high-density lipoprotein (HDL) cholesterol. The Friedewald’s formula was used to calculate low-density lipoprotein (LDL) cholesterol concentration [19]. Additionally, we calculated the level of non-HDL cholesterol by subtracting HDL cholesterol from TC and a very-low density lipoprotein (VLDL) level as a 20% of the TG level [20]. The ratios of TC/HDL, TG/HDL and LDL/HDL were also assessed.

In each patient and healthy subject the status of dyslipidaemia (either TC ≥ 200 mg/dL, or HDL < 40 mg/dL, or non-HDL ≥ 145 mg/dL) and hypertriglyceridemia (for children aged up to 9 years TG ≥ 100 mg/dL, and for children aged 10–19 years, TG ≥ 130 mg/dL) were established following the Sultan et al. [21] recommendations. The following intervals for lipid ratios were used: LDL/HDL normal < 3, borderline 3–4, and high > 4; TC/HDL normal < 4, borderline 4–5, and high > 5; TG/HDL normal < 3, and above normal > 3.

Our study has a retrospective character however the lipid levels were measured during the acute phase of AIS whereas in most of the headache children they were measured at the inter-ictal period.

### 2.3. Meta-Analysis

The following databases were searched: PubMed, Science Direct, Embase, and Google Scholar (last search in February 2021) with appropriate key words: “arterial ischemic stroke” or “stroke”, and “lipids” or “cholesterol”, and “children” for eligible articles. The meta-analysis was conducted according to the PRISMA statement (Preferred Reporting Items for Systematic Reviews and Meta-Analyses).

The titles and abstracts of the extracted articles were screened. The references cited in the found articles were also searched in order to identify other published articles on the topic. The first step of elimination was the duplication and clear irrelevance to the topic based on the article’s title. The remaining papers were comprehensively analyzed to determine whether they contained the relevant information.

Retrospective, and prospective case-control studies, with available levels of TC, LDL, HDL, and TG in both patients and controls were considered. The language was limited to English. The exclusion criteria were as follows: lack of lipid levels, design other than case-control study, and language other than English.

### 2.4. Statistical Analysis

#### 2.4.1. Case-Control Study

STATISTICA 13.0 software (STATSOFT; Statistica, Tulsa, OK, USA) was used to perform the statistical tests. The mean values (M) and standard deviations (SD) were estimated for continuous variables, while the absolute numbers (*n*) and relative numbers (%) were estimated for the categorical variables. The normality of distribution of quantitative data was evaluated by the Shapiro–Wilk *W* test. For the homogeneity of the variance Levene’s test was used. Comparisons of the mean values of quantitative data among all analyzed subgroups were performed with the use of the following tests: analysis of variance (ANOVA), when the distributions of data did not differ from the normal distribution, or the Kruskal–Wallis test, when the distributions of quantitative data did differ from the normal distribution or the assumption of homogeneity was violated. In the case of significant differences observed using the Kruskal–Wallis test, the Mann–Whitney *U* tests was used for post-hoc pairwise comparisons [2]. The Freeman-Halton extension of the Fisher exact test (VassarStats) was used to calculate differences in the prevalence of dyslipidemia and hypertriglyceridemia between analyzed subgroups. The value of *p* ≤ 0.05 was considered to be statistically significant. The odds ratio (OR) with a 95% confidence interval (CI) was calculated for the occurrence of hypertriglyceridemia or dyslipidemia in children with AIS compared to children with headache.

#### 2.4.2. Meta-Analysis

The Review Manager software (RevMan version 5.4 Cochrane, London, UK) was used to calculate standardized mean difference (SMD) together with a 95% confidence interval (CI) for the levels of TC, LDL, HDL, and TG in meta-analysis between children with AIS and controls. The results of the heterogeneity between studies, i.e., the *I*^2^ test at level of 50%, allowed to swapping between the random effects model and the fixed effects model. *I*^2^ expresses the proportion of dispersion due to heterogeneity and *I*^2^ at 25%, 50% and 75% was suggested as low, intermediate and high inconsistency, respectively.

## 3. Results

### 3.1. Characteristics of the Study Groups

Almost 65% of the patients suffered from AIS and 35%—from headache. In both patients subgroups as well as in controls male sex predominated, however the proportion of girls and boys did not differ between groups. Table 1 demonstrates the age and sex prevalence in all analyzed groups.

The median age of analyzed children differed significantly between groups. Children suffering from headache were oldest while children with AIS were youngest. The mean age was significantly higher in the headache subgroup compared to the AIS subgroup. In children with AIS 10% had posterior stroke.

### 3.2. Levels of Lipid Parameters in All Analyzed Groups

Mean concentrations of lipid parameters in all analyzed groups of children are shown in Table 2. The mean TC was significantly higher in children with AIS than in controls or in children with headache. The level of LDL was highest in controls and was significantly different from the LDL level in children with AIS. LDL concentration also differed between AIS children and children with headache. The mean level of HDL was highest in children with headache and differed from mean level of HDL in controls. However, there was no difference in HDL concentration between children with AIS and controls. In turn, mean levels of both TG and VLDL were significantly different between all groups. The non-HDL level was comparable between children with AIS and controls but it was significantly lower in children with headache compared to controls and AIS children. The TC/HDL ratio was highest in controls while the TG/HDL ratio was highest in the AIS subgroup (Table 2).

**Figure 1:**
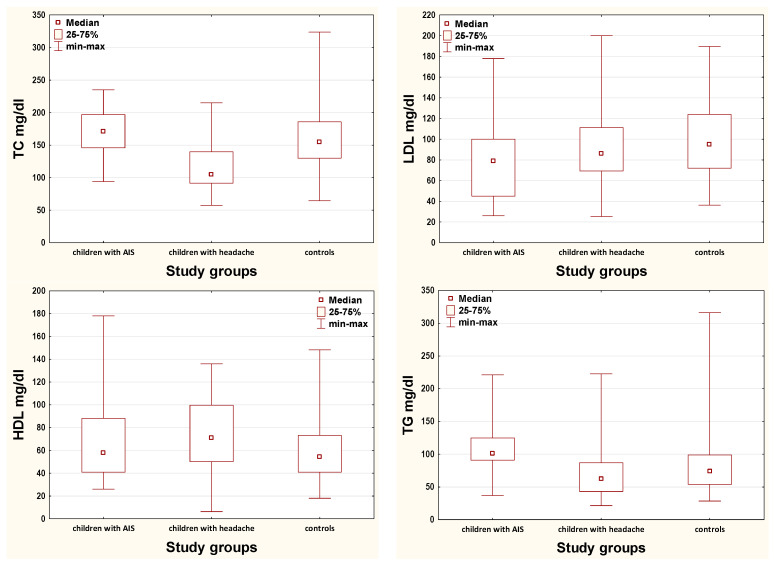
Box plots show median concentrations, interquartile range (the 25th to the 75th percentile of lipid parameters) and min-max. *p* values were determined by the Kruskal—Wallis test for comparison of three groups. For TC, HDL and TG parameters median test revealed statistical significance (TC: χ^2^ = 25.77, df = 2, *p* < 0.001; LDL: χ^2^ = 5.27, df = 2, *p* = 0.072; HDL: χ^2^ = 7.54, df = 2, *p* = 0.023; TG: χ^2^ = 55.28, df = 2, *p* < 0.001). AIS—arterial ischemic stroke; TC—total cholesterol; LDL—low density lipoprotein; HDL—high density lipoprotein; TG—triglycerides.

Median concentrations of TC, HDL, and TG differed significantly between children with AIS, children with headache and controls (Figure 1). In turn, median levels of LDL showed no significance between the three groups.

With respect to the lipid ratios, children with AIS, children with headache and control subjects had a similar prevalence of cases with a high value of TC/HDL ratio, whereas no child with headache had a borderline value of TC/HDL ratio. As for the LDL/HDL ratio no statistical significance was observed between studied analyzed groups. Similarly, the prevalence of subjects with a TG/HDL ratio above normal did not differ between the groups (Table 3).

**Figure 2:**
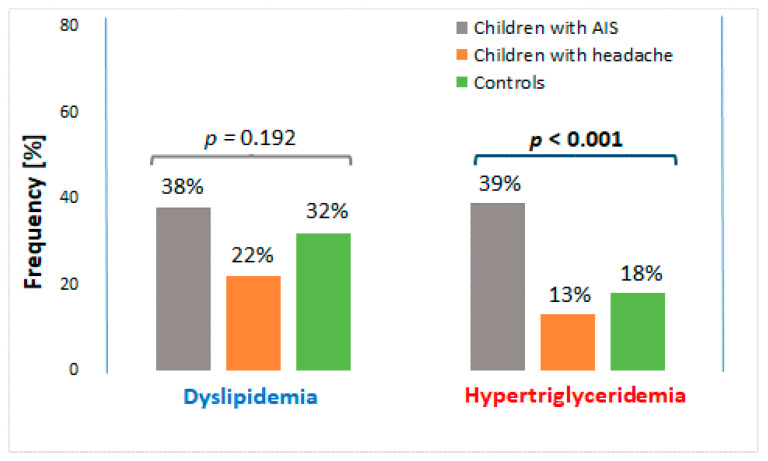
Prevalence of dyslipidemia and hypertriglyceridemia in all analyzed groups. AIS—arterial ischemic stroke. Significant difference is in bold.

Hypertriglyceridemia was the most prevalent in children with AIS, and least common in children with headache (*p* = 0.001) (Figure 2). In turn, a similar tendency was observed for dyslipidemia but the difference was not significant. The odds for a pediatric patients suffering from AIS of having hypertriglyceridemia was over 4-fold higher than for children with headache (OR = 4.16 95% CI 1.58–10.94, *p* =0.004). Similarly, the odds for a child with AIS of having dyslipidemia was over 2-fold higher than for a child with headache (OR = 2.13 95% CI 0.93–4.89, *p* = 0.078) although the difference was on the bounds of significance.

### 3.3. Lipid Levels in Young Patients with AIS—Meta-Analysis of Case-Control Studies

**Figure 3:**
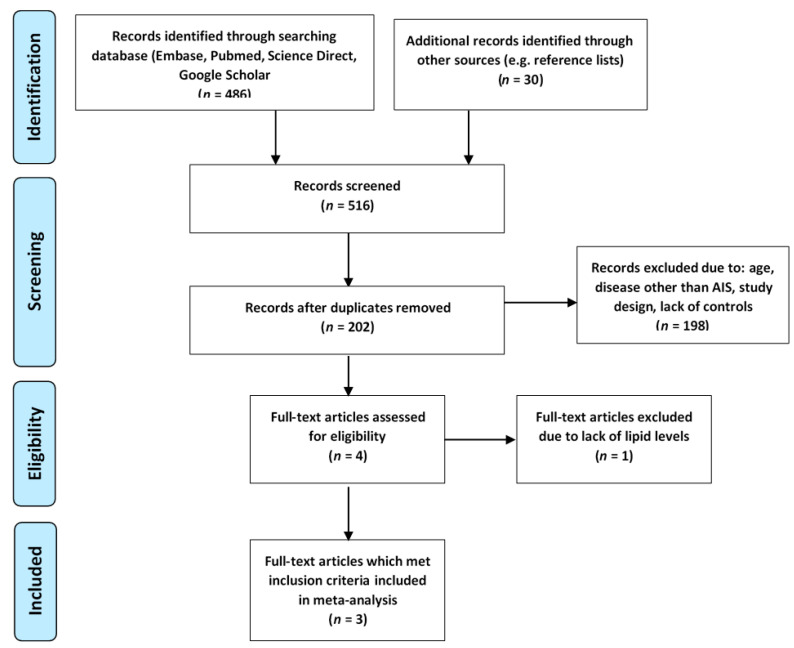
Flow chart presenting the process of searching for the eligible articles according to PRISMA guidelines.

Literature searching revealed four case-control studies based on children and adolescents with AIS, which analyzed concentrations of lipid parameters [22,23,24,25]. Figure 3 shows a flow diagram of the searching process.

The study by Leniček Krleža et al. [25] was excluded since no crude levels of lipids were demonstrated just counts and percentages for each lipid parameter. One of the studies analyzed young patients together with adolescents [23] and we decided to include the results. Finally, meta-analysis was based on three published case-control studies and the results obtained in the present analysis.

**Figure 4:**
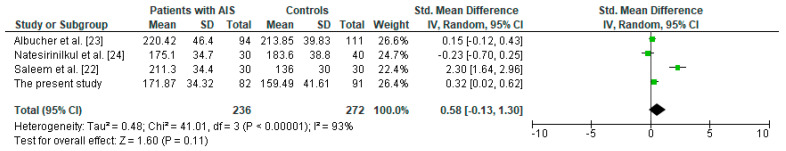
Forest plot for TC level in the groups of pediatric patients with AIS and controls [22,23,24]. TC—total cholesterol; AIS—arterial ischemic stroke; CI—confidence interval; *I*^2^—heterogeneity; df—degrees of freedom.

**Figure 5:**
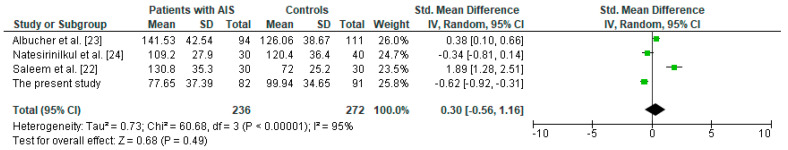
Forest plot for LDL level in the groups of pediatric patients with AIS and controls [22,23,24]. LDL—low density lipoprotein; AIS—arterial ischemic stroke; CI—confidence interval; *I*^2^—heterogeneity; df—degrees of freedom.

**Figure 6:**
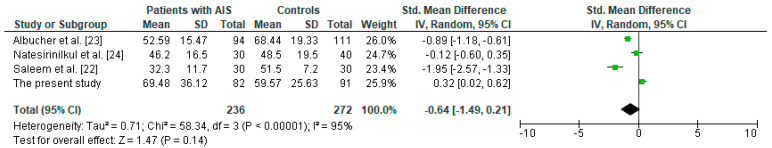
Forest plot for HDL level in the groups of pediatric patients with AIS and controls [22,23,24]. HDL—high density lipoprotein; AIS—arterial ischemic stroke; CI—confidence interval; *I*^2^—heterogeneity; df—degrees of freedom.

**Figure 7:**
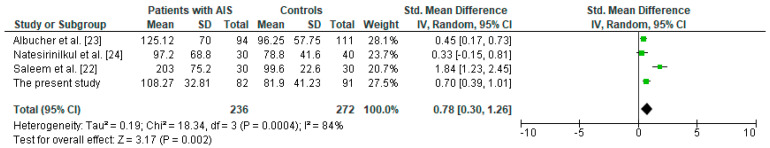
Forest plot for TG level in the groups of pediatric patients with AIS and controls [22,23,24]. TG—triglycerides; AIS—arterial ischemic stroke; CI—confidence interval; *I*^2^—heterogeneity; df—degrees of freedom.

The results of the carried out meta-analyses were summarized using forest plots (Figure 4, Figure 5, Figure 6 and Figure 7). In all analyses no heterogeneity was observed, thus a random effects model was used to calculate SMD.

The meta-analysis was conducted on a total number of 236 young patients with AIS and 272 healthy controls. Patients were examined within a wide range of age (from 0 to 45 years of age). It was demonstrated that young patients with AIS had significantly higher levels of TG than controls. The significant SMD was found for the comparison of triglycerides level between patients and controls (0.78 95%CI 0.30–1.26 *p* = 0.002). The conversion of SMD into OR gave the value of 1.41 which suggests that TG level may increase the odds for AIS almost 1.5-fold. For total cholesterol SMD was close to the significance level (*p* = 0.11). In turn, no significant SMD was observed for HDL and LDL levels (*p* = 0.14 and *p* = 0.49, respectively).

## 4. Discussion

In the present study mean values of TC, HDL, TG, and VLDL differed significantly between children with AIS and controls. Except for LDL levels, there were significant differences in the levels of remaining lipid parameters between children with headache and controls. Moreover, only LDL level did not differentiate children with AIS from children with headache.

Increased levels of lipid parameters (i.e., TC, LDL and TG) are one of the main causes of cardiovascular and cerebrovascular disease in adults. In pediatric patients the major and most common underlying problem in AIS pathogenesis is heart defects and the associated surgical procedures. In these patients the embolic mechanism is undoubtedly the dominant factor leading to AIS. However, due to the complex nature of childhood AIS, the heart defect in a child does not exclude the presence of other risk factors, e.g., lipid disturbances.

There are limited data on the role of disturbances of lipid metabolism in the etiopathogenesis of AIS in children and the results provided are ambiguous. This is mainly due to the size of the studied groups and the differences in age of the patients. In the present study, in addition to case-control analysis, we performed meta-analysis based on the present results as well as on the available data on lipid levels in children and adolescents. A meta-analysis enables pooling data from a smaller inconclusive studies and yields greater statistical power. Therefore, the problem of smaller groups of both cases and controls may be overcome and a single best estimate may be generated.

Similarly to our results, the study by Saleem et al. [22] demonstrated that children with AIS had significantly higher concentration of TC, LDL, HDL and TG compared to healthy children. In addition, the authors observed a positive correlation between plasma homocysteine level and both LDL and TG levels (r = 0.34, *p* = 0.03 and r = 0. 4, *p* = 0.01, respectively) [22]. On the other hand, the study by Natesirinilkul et al. [24] did not observe higher lipid levels in children with AIS compared to controls. Previously, we have demonstrated that lipid parameters were comparable between children with AIS and diagnosed with focal cerebral arteriopathy of childhood (FCA) and children with AIS but without FCA [26]. Additionally, in the study by Albucher et al. [23], mean level of TC did not differ between young patients with AIS and controls however mean concentrations of LDL, VLDL and TG were significantly higher in the study cases than in healthy subjects. In turn, in the young patients aged 25 to 39 years, low HDL emerged as a significant risk factors for AIS, while a high level of LDL was found to be inversely associated with the disease [27]. Significantly higher levels of TC and TG and a significantly lower level of HDL were also observed in young Chinese patients with AIS, compared to healthy cases [28]. The levels of LDL were comparable between the studies [28].

In the analyzed group of AIS children, the frequencies of dyslipidemia and hypertriglyceridemia were comparable—38% and 39%, respectively. The prevalence of hypertriglyceridemia in AIS children was significantly higher compared to children with headache and controls. Our results are comparable to data published previously. In the study from International Pediatric Stroke Study (IPSS), 41% of the children with AIS had elevated level of TG and dyslipidemia was observed in 36% cases [21]. The study by Reuter et al. [14] based on 1243 healthy children and adolescents from Brazil, demonstrated that dyslipidemia was present in 42% of cases, with higher prevalence in girls than in boys. On the contrary, a study based on Thai children with AIS demonstrated that hypercholesterolemia was present in 20% of patients while hypertriglyceridemia was present in 26.7% of cases and did not significantly differ from the controls [24]. In very young adults with AIS, aged 18–35 years, dyslipidemia was observed in 37% of them while hypertension was present only in 9% of cases [29]. On the other hand, metabolic disorders, including hyperlipidemia were observed in 2.3% of Taiwanese children with ischemic stroke in a nationwide population study [30]. Interestingly, the study by Bigi et al. [31] demonstrated that hyperlipidemia concerned 11 out of 128 children with AIS (aged up to 16 years), as well as 54 young adults (aged 16–45 years), out of 199 analyzed patients and the difference was statistically significant. Seven children, out of those having hyperlipidemia presented hypercholesterolemia [31].

Published data on pediatric AIS include children in a wide range of age from birth to 16–19 years old, which may influence the results. Previously, Rambaud et al. pointed out that AIS in adolescents showed both pediatric and adult features [32].

In the present study, we also analyzed lipid levels in children with headache since the association of migraine with cardiovascular disease was previously suggested. Tana et al. [33] demonstrated a significant positive association between migraine frequency and intensity with levels of total cholesterol and LDL cholesterol. The authors observed that patients with migraine without aura (mean age 36.69 ± 12.35 years) had slightly, but not significantly higher levels of TC and LDL compared to migraineurs with aura, who were older [33].

Babayan et al. [34] noticed that patients with migraine showed greater vasomotor reactivity and more often declared a positive family history for cardiovascular disease. It may be associated with similar genetic factors that are involved in the development of cardiovascular diseases and migraines [34].

Migraine pathophysiology is not fully understood, but neurovascular disorders seem to be one of the most important mechanisms involved in headache. During migraine attack the dilatation of the brain blood vessels is observed especially in arachnoid matter. Inflammation develops as a result of the release of neuropeptides from the nerve endings into the perivascular space. The brainstem, an integral part of the nociceptive system, also plays an important role in migraine pathomechanism through the neuronal centers responsible for the conversion of vascular and neuronal activity (CSD—cortical spreading depression) [35,36]. Changes in the concentration of some factors released under the influence of hypoxia or in thrombotic changes, e.g., sCD40 L and PAI-1 were also observed.

The results of our meta-analysis suggest that a higher level of TG may increase the risk for AIS (OR = 1.41). It is in agreement with the general trend in pediatrics, which emphasizes that increased levels of TG (and decreased HDL concentrations) are a risk factor for cardiovascular complications [37,38]. Earlier, triglycerides were reported to correlate significantly with PAI-1 antigen levels in adult patients with stroke [39]. In turn, recent data on a sizeable group of patients with AIS proved that triglycerides may be considered as risk factor for ischemic stroke and not for hemorrhage stroke [40]. On the other hand, our meta-analysis showed no relations between LDL and HDL levels and AIS, but the SMD for TC was close to the statistical significance.

The present meta-analysis of lipid levels in children with AIS may be burdened with an error resulting from the wide age range of patients from the studies included. The study by Saleem et al. [22] included children with AIS with an age range from 0.33 to 18 years (adolescents accounted for 33%). In the study by Natesirinilkul et al. [24] children with AIS were aged from 1 to 18 years old. On the other hand, in the study by Albucher et al. [23] young adults were also analyzed apart from adolescents. However, we did not find another case-control study analyzing the levels of all lipid parameters in children or adolescents suffering from AIS. Therefore further data are needed to confirm the pilot association.

The present study has several limitations. Its retrospective design hinders the access to some data which might be important for analysis. It is based on a relatively small stroke sample size which results from the rarity of AIS in children. We also included a low number of patients with headache. Moreover, the age of children with AIS was lower compared to children with headache. Another limitation of the study is the lack of analysis of the correlation between the concentrations of lipids and the child’s weight and BMI. In our previous paper, a positive correlation between these parameters was found in children with headache [17].

## 5. Conclusions

The obtained results, i.e., the higher frequency of dyslipidemia in children with AIS compared to children with headaches, may be a significant contribution to the further research on the role of lipid metabolism disorders in the etiopathogenesis of childhood stroke. Previous studies in this field, conducted on relatively small groups of patients, yield ambiguous results. It seems that the disorders underlying the ischemic and vascular changes in the central nervous system appear already in childhood. An expression of this pathomechanism may be lipid disorders observed in children with AIS and headaches. It seems that abnormalities operating at the cellular and tissue levels can be reflected in everyday clinical practice with the help of available and inexpensive laboratory lipid examination. Extending research on the importance of dyslipidemia in AIS would also be of practical importance, as dietary and pharmacological actions in a child after AIS could be treated as a secondary prevention of subsequent incidents of cerebral ischemia.

However, further case-control prospective studies are needed to confirm such relationships and to establish the specific cut-off points of the selected laboratory analyzes.

## Figures and Tables

**Table 1:** Characteristics of age and sex in subgroups of patients with AIS and headache as well as in controls.

	Children with AIS *n* = 82 (64.57%)	Children with Headache *n* = 45 (35.43%)	Controls *n* = 91	p *	p **	p ***
Age (years),M ± SD	8.52 ± 5.73 ^a^	11.90 ± 3.82	10.38 ± 4.65	0.059	0.128	**0.001**
Median, (Min-Max)	8 (0.11–18);	11 (7–18);	11 (0.8–21);			
Interquartile range (IQR)	11 (3–14)	7 (8.5–15.5)	6.5 (7–13.5)			
Sex, *n* (%)				0.671	0.862	0.603
Girls	29 (35.37)	18 (40.00)	35 (38.50)			
Boys	53 (64.63)	27 (60.00)	56 (61.50)			

AIS—arterial ischemic stroke; M—mean; SD—standard deviation; IQR—interquartile range; ^a^—age at stroke onset; *—AIS subgroup vs controls; **—headache vs controls; ***—AIS subgroup vs headache; significant differences are in bold.

**Table 2:** Levels of lipid parameters in all analyzed pediatric groups.

Lipid Parameters	AIS Subgroup *n* = 82	Headache Subgroup *n* = 45	Controls *n* = 91	p *	p **	p ***
TC (mg/dL), M ± SD	171.87 ± 34.32	118.13 ± 38.39	159.49 ± 41.61	**0.016**	**<0.001**	**<0.001**
LDL (mg/dL), M ± SD	77.65 ± 37.39	91.10 ± 34.35	99.94 ± 34.65	**<0.001**	0.183	**0.041**
HDL (mg/dL), M ± SD	69.48 ± 36.12	71.94 ± 35.77	59.57 ± 25.63	0.295	**0.016**	0.399
TG (mg/dL), M ± SD	108.27 ± 32.81	70.57 ± 37.96	81.90 ± 41.23	**<0.001**	**0.049**	**<0.001**
VLDL (mg/dL), M ± SD	21.65 ± 6.56	14.11 ± 7.59	16.38 ± 8.25	**<0.001**	**0.049**	**<0.001**
Non-HDL (mg/dL), M ± SD	103.74 ± 41.90	54.32 ± 44.86	104.23 ± 45.00	0.692	**<0.001**	**<0.001**
TC/HDL, M ± SD	3.09 ± 1.58	2.83 ± 3.53	3.18 ± 1.76	0.637	**<0.001**	**<0.001**
LDL/HDL, M ± SD	1.61 ± 1.23	2.12 ± 2.88	1.98 ± 1.28	**0.033**	**0.013**	0.332
TG/HDL, M ± SD	1.99 ± 1.16	1.90 ± 3.33	1.79 ± 1.96	**0.030**	**0.019**	**<0.001**

AIS—arterial ischemic stroke; M—mean; SD—standard deviation; IQR—interquartile range; *—comparison AIS subgroup vs. controls; **—comparison headache vs controls; ***—comparison AIS subgroup vs. headache; significant differences are in bold.

**Table 3:** Prevalence of lipid ratios’ intervals in analyzed groups.

Lipid Parameters		AIS Subgroup *n* = 82	Headache Subgroup *n* = 45	Controls *n* = 91	*p*
TC/HDL, *n* (%)	Normal <4	59 (73)	39 (87)	74 (81)	**0.007**
	Bordeline 4–5	14 (17)	0 (0)	7 (8)	
	High >5	8 (10)	6 (13)	10 (11)	
LDL/HDL, *n* (%)	Normal <3	72 (89)	37 (82)	79 (86)	0.799
	Bordeline 3–4	5 (6)	3 (7)	6 (7)	
	High >4	4 (5)	5 (11)	6 (7)	
TG/HDL, *n* (%)	Normal <3	68 (84)	4 (89)	81 (89)	0.565
	Above normal >3	13 (16)	5 (11)	10 (11)	

Significant difference is in bold.

## Data Availability

The data which we presented in the study are available from authors on request. The data are not publicly available due to privacy restrictions.
